# A Combination of an Antimitotic and a Bromodomain 4 Inhibitor Synergistically Inhibits the Metastatic MDA-MB-231 Breast Cancer Cell Line

**DOI:** 10.1155/2019/1850462

**Published:** 2019-12-11

**Authors:** Thandi Mqoco, André Stander, Anna-Mart Engelbrecht, Anna M Joubert

**Affiliations:** ^1^Department of Physiology, University of Pretoria, Private Bag X323, Gezina, Pretoria 0031, South Africa; ^2^Department of Physiological Sciences, Stellenbosch University, Stellenbosch, South Africa

## Abstract

Current chemotherapeutic agents have many side effects and are toxic to normal cells, providing impetus to identify agents that can effectively eliminate tumorigenic cells without damaging healthy cells. The aim of this study was to examine whether combining a novel BRD4 inhibitor, ITH-47, with the antimitotic estradiol analogue, ESE-15-ol, would have a synergistic effect on inhibiting the growth of two different breast cancer cell lines *in vitro*. Our docking and molecular dynamics studies showed that compared to JQ1, ITH-47 showed a similar binding mode with hydrogen bonds forming between the ligand nitrogens of the pyrazole, ASN99, and water of the BRD4 protein. Data from cell growth studies revealed that the GI_50_ of ITH-47 and ESE-15-ol after 48 hours of exposure was determined to be 15 *μ*M and 70 nM, respectively, in metastatic MDA-MB-231 breast cancer cells. In tumorigenic MCF-7 breast cancer cells, the GI_50_ of ITH-47 and ESE-15-ol was 75 *μ*M and 60 nM, respectively, after 48 hours of exposure. Furthermore, the combination of 7.5 *μ*M and 14 nM of ITH-47 and ESE-15-ol, respectively, resulted in 50% growth inhibition of MDA-MB-231 cells resulting in a synergistic combination index (CI) of 0.7. Flow cytometry studies revealed that, compared to the control, combination-treated MDA-MB-231 cells had significantly more cells present in the sub-G_1_ phase and the combination treatment induced apoptosis in the MDA-MB-231 cells. Compared to vehicle-treated cells, the combination-treated cells showed decreased levels of the BRD4, as well as c-Myc protein after 48 hours of exposure. In combination, the selective BRD4 inhibitor, ITH-47, and ESE-15-ol synergistically inhibited the growth of MDA-MB-231 breast cancer cells, but not of the MCF-7 cell line. This study provides evidence that resistance to BRD4 inhibitors may be overcome by combining inhibitors with other compounds, which may have treatment potential for hormone-independent breast cancers.

## 1. Introduction

Breast cancer is a global health problem [1, 2] and is predicted to increase by 78% in 2030 [3]. Currently, researchers are trying to identify and develop compounds that are effective against malignant cells without damaging healthy cells [4]. The expression of genes in tumour cells and subsequent proliferation of these cells are potential targets for drug discovery.

Much research has focused on the myelocytomatosis (MYC) gene, which is amplified in multiple types of cancer [5]. The MYC, or c-Myc, gene is a key regulator of cell growth and apoptosis [6]; deregulated expression of c-Myc is known to stimulate uncontrolled cell proliferation [7]. The expression of the c-Myc gene depends on the presence of bromodomain-containing protein 4 (BRD4), which is encoded by the BRD4 gene. Subsequently, inhibiting or blocking the BRD4 protein from gaining access to acetylated histones may prevent the transcription of c-Myc and thus limit the proliferation of tumour cells [8, 9].

One such inhibitor, JQ1 is a nonselective BRD4 inhibitor which binds competitively to the acetyl-lysine recognition pocket of the bromodomain and extra-terminal domain (BET) resulting in the displacement of BET proteins from chromatin [8, 10]. This prevents the expression of genes such as the c*-Myc* oncogene and the proapoptotic Bcl2 protein [8, 9]. Although it is an effective inhibitor, JQ1 has a short half-life, and thus, it is important to develop analogues of this compound. The newly designed benzotriazepine compound, ITH-47, is a BRD4-selective inhibitor that shows effective antiproliferative activity against U-937 leukemia cells, as well as synergistic inhibition when combined with the antiglycolytic compound, 3-bromopyruvate [11]. ITH-47 was shown to be more than 2x selective against the cancer-associated BRD4 (490 nM) protein when compared to BRD2 (1120 nM) [11].

Aside from blocking the expression of certain genes, arresting mitosis is also an effective avenue for treating cancer [12]. Included in the group of mitosis inhibitors are microtubule-targeting compounds such as paclitaxel, epothilones, 2-methoxyestradiol (2ME2), and podophyllotoxin [13, 14]. These compounds are divided into two groups depending on their binding site on the microtubules [13] Disrupting the normal functioning of the mitotic spindle causes mitotic arrest and subsequent cell death [13–16]. Although these compounds are highly effective chemotherapeutic drugs, bioavailability is an important challenge [17]. Thus, much research aims at identifying more effective microtubule-targeting agents.

One such microtubule-targeting compound is 2-ethyl-3-*O*-sulphamoyl-estra-1,3,5(10),15-tetraen-17-ol (ESE-15-ol) which is an analogue of 2ME2. 2ME2 is a metabolite of 17-*β*-estradiol with antitumor and antiangiogenic properties both *in vitro* and *in vivo* [18]. Previous studies in our laboratory have shown that ESE-15-ol is more potent than 2ME2 and that ESE-15-ol inhibits cell growth of the human tumorigenic breast epithelial cell line (MCF-7), human metastatic breast cell line (MDA-MB-231), human cervical adenocarcinoma cells (HeLa), and human nontumorigenic breast epithelial cell line (MCF-12A) [19, 20]. ESE-15-ol binds to the colchicine binding site on tubulin, thus triggering cells to undergo mitotic arrest which consequently leads to the induction of apoptosis [19, 20]. The MCF-12A cells were the least affected by 50 nM ESE-15-ol when compared to MDA-MB-231 and MCF-7 cells [19]. The antitumor activity of ESE-15-ol was displayed in breast cancer (MDA-MB-231 and MCF-7) cells by inducing mitochondrial membrane depolarization, abrogating the phosphorylation status of B-cell lymphoma protein 2 (Bcl-2) and by affecting the expression of genes linked with cell death and mitosis [19].

The use of combination chemotherapeutic regimens that exert their chemotherapeutic effects via different mechanisms of action has been a pertinent step in the improvement of cancer treatment; such chemotherapeutic regimens may improve the efficacy of single-agent treatment regimens [21–23]. Improvement of the efficacy of treatment is achieved by targeting different pathways such that the sum of the effects of individual drugs is greater than it would have been for the individual drugs [22]. Moreover, combination drug regimens have the potential to synchronously reduce drug resistance and enhance drug-tumour interactions causing a reduction in tumour size and/or induce apoptosis [22].

**Figure 1:**
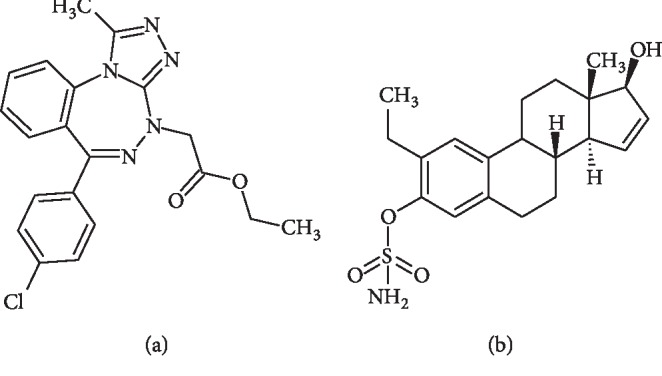
Chemical structure of (a) ethyl-2-[6-(4-chlorophenyl)-1-methyl-4H-benzo[e][1,2,4]triazolo[3,4-c][1,2,4]triazepin-4 yl] acetate (ITH-47) and (b) 2-ethyl-3-*O*-sulphamoyl-estra-1,3,5(10),15-tetraen-17-ol (ESE-15-ol) (ACD/ChemSketch Freeware version 12.0).

In this study, we investigated whether a combination of two novel *in silico*-designed compounds, namely, ITH-47 (BRD4 inhibitor) and ESE-15-ol (an antimitotic agent), would synergistically inhibit the growth of breast cancer cells, MCF-7 and MDA-MB-231 (Figure 1). We compared the docking behaviour of ITH-47 with the well-known JQ1, using molecular dynamics methods. We used crystal violet assays to assess the effect of ESE-15-ol and ITH-47 on cell growth and flow cytometry to assess cell cycle progression and apoptosis.

## 2. Materials and Methods

### 2.1. Cell Lines

The MCF-7 cell line is a human tumorigenic adherent breast epithelial cell line that expresses the oestrogen receptor-alpha (ER-*α*) as well as the androgen, progesterone, and glucocorticoid receptors [24]. The MDA-MB-231 cell line is an aggressive and poorly differentiated breast cancer (triple-negative breast cancer, TNBC) which expresses oestrogen receptor (ER), progesterone receptor (PR), and human epidermal growth factor receptor 2 (HER2) negative [25]. Both cell lines are commercially available from Cellonex (Pty) Ltd. (Ferndale, Randburg, South Africa). Cells were grown and maintained in 25 cm^2^ tissue culture flasks in a humidified atmosphere at 37°C and 5% CO_2_. Cells were propagated in Dulbecco's modified Eagle's medium (DMEM) supplemented with 10% heat-inactivated fetal calf serum, 100 U/ml penicillin G, 100 *μ*g/ml streptomycin, and fungizone (250 *μ*g/l).

### 2.2. Compounds and Reagents

ESE-15-ol and ITH-47 were *in silico*-designed in our laboratory and are therefore not commercially available. These compounds were synthesized by iThemba Pharmaceuticals (Pty) Ltd (Modderfontein, Gauteng, South Africa) and by Wuxi Apptec (Shanghai, China). Stock solutions of 10 mM of ESE-15-ol and 50 mM of ITH-47 were dissolved in dimethyl sulfoxide (DMSO) and stored at −20°C. The DMSO volume in the final dilutions never exceeded 0.05% (v/v). The positive control for apoptosis induction included cells treated with vinblastine (0.2 *μ*M). All other chemicals, culture media, and reagents were bought from Sigma-Aldrich Co. (St Louis, United States of America). All plastic consumables were purchased from Lasec (Pty) Ltd. (Randburg, Johannesburg, South Africa).

### 2.3. Molecular Dynamics and Calculation of Binding Energy

To compare the binding energy of ITH-47 and JQ1, we performed molecular dynamics simulations using GROMACS 5.1.2 [26] and the AMBER force field [27] as described by Deepak et al. [28]. Briefly, 20 ns simulations with the docked pose of ITH-47 and JQ1 into BRD2(1), BRD2(2), BRD4(1), and BRD4(2) in saline water were performed after which the binding energy was calculated using the g_mmpbsa package with the molecular mechanics Poisson–Boltzmann surface area (MM-PBSA) software from the Gromacs and APBS packages [29, 30]. The angles and bond lengths of the triazolo and benzotriazepine were modified to reflect the angles and bonds of the Bzt compound 08K from 3U5L.

### 2.4. Cell Growth

Dose-dependent studies were conducted to investigate the antiproliferative effects of both ITH-47 and ESE-15-ol, as single agents and in combination, on tumorigenic epithelial (MCF-7) and metastatic (MDA-MB-231) breast cancer cell lines. The selection of concentrations (6.25–150 *μ*M ITH-47 and 50–150 nM ESE-15-ol) and times of exposure used in this study were based on previous studies conducted in our laboratory which showed the optimal antiproliferative activity of these compounds at chosen concentrations (data not shown). The 50% growth inhibitory (GI_50_) effect of ITH-47 on MDA-MB-231 and MCF-7 cells was calculated according to the National Cancer Institute [31].

Crystal violet staining is a triphenylmethane dye also known as gentian violet. Crystal violet is a simple, reliable, and quick method to test for cell viability [32]. When cells die, they lose the ability to adhere, and thus, they detach from the cell culture plates and they are lost from the population of cells [33]. Crystal violet is a dye that binds to both proteins and DNA of viable cells [33, 34]. In this *in vitro* study, a crystal violet staining assay was used to determine the effects of ESE-15-ol and ITH-47 on cell viability. The absorbance of the dye measured at 570 nm corresponds to cell quantities.

Cells (5,000 per well) were seeded in 96-well tissue culture plates and incubated for 24 hours to ensure attachment. Following incubation, DMEM was discarded and cells were treated to a dilution series of ESE-15-ol and ITH-47, in isolation and in combination. To stop the experiment, 100 *μ*l of 1% glutaraldehyde in a water solution was added to each well for a period of 15 minutes. Then, crystal violet (100 *μ*l) (1%, prepared in water) was added to the wells which were incubated at room temperature for 30 minutes. Following incubation, the culture wells were submerged in running tap water for 15 minutes. Plates were placed on a paper towel to dry overnight. The next day, the crystal violet was dissolved by adding 200 *μ*l of Triton-X100 (0.2% in water) to each well. Plates were incubated for 30 minutes at room temperature, and then, 100 *μ*l of the liquid content was transferred to 96-well plates. The absorbance of the samples was determined using an ELX800 Universal Microplate Reader (Bio-Tek Instruments, Inc., Analytical Diagnostic Products, Weltevreden, South Africa).

### 2.5. Drug Combination Calculations

To assess possible synergism between ITH-47 and ESE-15-ol on inhibiting cell growth of MDA-MB-231 cells, the combination index (CI) was calculated using the results of the crystal violet assay. The CI was calculated using the following equation [35]:

(1) $$\begin{array}{c} \text{CI}=\frac{{\left(D\right)}_{1}}{{\left(D_{x}\right)}_{1}}+\frac{{\left(D\right)}_{2}}{{\left(D_{x}\right)}_{2}}, \end{array}$$

where (*D*)_1_ and (*D*)_2_ are the concentrations of ITH-47 and ESE-15-ol that exhibit 50% cell growth inhibition when applied simultaneously to the cells whereas (*D*_*x*_)_1_ and (*D*_*x*_)_2_ are the concentrations of ITH-47 and ESE-15-ol that exhibit 50% cell growth inhibition when used in isolation [35].

The CI values indicate a synergistic effect when <1, an antagonistic effect when >1, and an additive effect when equal to 1 [35].

### 2.6. Flow Cytometry: Cell Cycle Progression

PI is a fluorescent stain that intercalates into the DNA, and thus, the fluorescence thereof will increase proportionally to the increase in the DNA content of a sample [36]. Flow cytometry utilising PI staining enables the quantification of cells in the various cell cycle phases based on the DNA content of each cell [36]. In this study, the effects of ITH-47 and ESE-15-ol, as single agents and in combination, on cell cycle progression were analysed using flow cytometry.

Exponentially growing cells (500,000 cells per 25 cm^2^ flask) were seeded and incubated overnight to allow for attachment onto the flask. Following attachment, cell growth medium was replaced and the cells were treated with test compounds in combination and as single agents (7.5 *μ*M ITH-47 and/or 14 nM ESE-15-ol) and compared to relevant controls. After the treatment period, the cells were subcultured and the pellet was resuspended in 1 ml of fresh cell growth medium. Thereafter, 500,000 cells per sample were centrifuged for 10 minutes at 300 × *g*. Subsequently, the medium was discarded and the pellet was resuspended in 200 *μ*l of chilled PBS containing 0.1% FCS. Then, 4 ml of chilled ethanol (70%) was added dropwise while vortexing the cells, thus preventing cells from clumping to each other. Thereafter, the cells were stored in the fridge for 24 hours. Following 24 hours, the cells were centrifuged at 300 × *g* for 10 minutes. The supernatant was discarded and cells were resuspended in 1 ml of PBS consisting of 40 *μ*g/ml PI as well as 100 *μ*g/ml RNase A. The solution was kept in an incubator for 45 minutes (5% CO_2_, 37°C). The fluorescence of PI was quantified by means of a FC500 System flow cytometer (Beckman Coulter South Africa (Pty) Ltd.). For analysis, data from at least 10,000 events were analysed with CXP software (Beckman Coulter South Africa (Pty) Ltd. (Pretoria, Gauteng, South Africa). Cell cycle distributions were analysed using Cyflogic 1.2.1 (Perttu Terho & Cyflo Ltd) by assigning relative DNA content per cell to sub-G_1_, G_1_, S, and G_2_/M fractions.

### 2.7. Flow Cytometry: Apoptosis

Apoptotic cells have exposed phosphatidylserine molecules that bind annexin V, while necrotic cells have compromised membranes and absorb PI [37]. Cells (500,000) were seeded in a 25 cm^2^ flask and then incubated overnight to allow for attachment. Following incubation, the medium was removed and the cells were exposed to 7.5 *μ*M of ITH-47 and 14 nM of ESE-15-ol as single agents and in combination and with appropriate controls. After exposure, cells were passaged and 500,000 cells were mixed in 1x binding buffer (1 ml) and then centrifuged at 300 × *g* for 10 minutes (Hermle Z 306 centrifuge, Labnet, South Africa). The supernatant was removed and then the cells were mixed in 100 *μ*l of the 1x binding buffer. Then, we added 10 *μ*l of annexin V-FITC and incubated the solution in the dark for 15 minutes at room temperature according to the supplier's manual. Following incubation, the cells were washed in 1 ml of the 1x binding buffer and then centrifuged at 300 × *g* for 10 minutes. The supernatant was discarded and cells were resuspended in 500 *μ*l of the 1x binding buffer solution. Before analysing the samples, 12.5 *μ*l of the PI (40 *μ*g/ml) was added and mixed into the solution. PI fluorescence and annexin V-FITC fluorescence were measured using a FC500 System flow cytometer (Beckman Coulter South Africa (Pty) Ltd) equipped with an air-cooled argon laser excited at 488 nm. Data from at least 10,000 events were analysed using CXP software (Beckman Coulter South Africa (Pty) Ltd. Data were further analysed using Cyflogic 1.2.1 software; data from fluorescence channel for green monomers (Fl1 log) and fluorescence channel for red monomers (Fl3 log) were represented as dot plots on the *y*- and *x*-axis in that order.

### 2.8. Spectrophotometry: Caspase 6 Colorimetric Assay

Caspases are a family of cysteine proteinases which are instrumental to the execution of apoptosis [38]. Apoptotic caspases belong to one of two categories: initiator caspases or effector/executioner caspases. Effector caspases include caspases 3, 6, and 7 which execute apoptotic cell death [39].

One million cells were seeded in 25 cm^2^ flasks. After 24 hours of incubation in an incubator (37°C, 5% CO_2_), cells were exposed to test compounds as single agents and in combination, as well as to the relevant controls. Thereafter, the cells were passaged and centrifuged for 10 minutes at 300 × *g* (Hermle Z 306 centrifuge, Labnet, South Africa). The cell pellets were mixed in 50 *μ*l of cell lysis buffer and kept on ice for 10 minutes. Subsequently, the cells were centrifuged at 300 × *g* for 10 minutes. After centrifugation, protein concentration was quantified using the BCA protein assay (Thermo Scientific, Johannesburg, South Africa). Then, the supernatant was added together with 50 *μ*l of 2X reaction buffer containing 10 mM dithiothreitol (DTT) and 5 *μ*l of 4 mM Ac-Leu-Glu-His-Asp-p-nitroanilide (*VEID-pNA*) was mixed with the supernatants and the reaction mixtures were kept in an incubator for 2 hours. After the incubation period, absorbance was read at 405 nm using an ELx800 Universal Microplate Reader available from Bio-Tek Instruments Inc. (Vermont, United States of America).

### 2.9. Protein Expression

Expression of the BRD4 and c-Myc proteins was analysed by western blot analysis. MDA-MB-231 and MCF-7 cells were treated with 7.5 *μ*M of ITH-47 and or 14 nM of ESE-15-ol for 48 hours. Following 48 hours, the cells were lysed and scraped and protein concentration was quantified using the Pierce® BCA protein assay kit (Thermo Fisher Scientific Inc., Rockford, Illinois, USA). Samples were diluted in loading buffer with *β*-mercaptoethanol and NuPAGE LDS buffer (1 : 4) (Sigma-Aldrich, St. Louis, USA)), boiled for 10 minutes, and subjected to gel electrophoresis. Each lane was loaded with 30 *μ*g of cell protein. Proteins were electrophoretically transferred onto polyvinylidene difluoride (PVDF) membranes overnight at 30 V. Immunolabeling was achieved by blocking the gel with 2% bovine serum albumin (BSA) for 1 hour at room temperature, rotating the gel overnight at 4°C with primary antibodies diluted (1 : 1000 BRD4; 1 : 10000 c-Myc) in 0.2% PBS-Tween and 2% BSA and subsequently incubating the membrane with a secondary goat anti-mouse/anti-rabbit horseradish antibodies at a dilution of 1 : 10,000 for 1 hour. Proteins were visualised using enhanced chemiluminescence substrate and the Biorad Chemidoc MP system (Bio-Rad Laboratories, Inc. California, United States of America). Bands were quantified using Image Lab software developed by Bio-Rad Laboratories, Inc. (California, United States of America) and BRD4/c-Myc: actin ratios were calculated.

### 2.10. Statistical Analysis

All experiments were conducted in triplicate. Mean values are presented in bar charts with T-bars representing standard deviations. Each independent crystal violet staining experiment had a technical repeat of 6. Differences between the different treatments were analysed using one-way analysis of variance (ANOVA) with Bonferroni correction and Student's *t*-test using GraphPad Prism 5.0 (GraphPad Inc.). Treatments were statistically significant if *P* < 0.05 and are indicated by an asterisk (^*∗*^). Flow cytometry analysis was carried out using data from at least 10,000 events that were repeated thrice. Bands produced in western blots were quantified using Image Lab developed by Bio-Rad Laboratories, Inc. (California, United States of America) and ratios of BRD4/c-Myc to actin were determined.

## 3. Results

### 3.1. Molecular Dynamics and Calculation of Binding Energy

**Figure 2:**
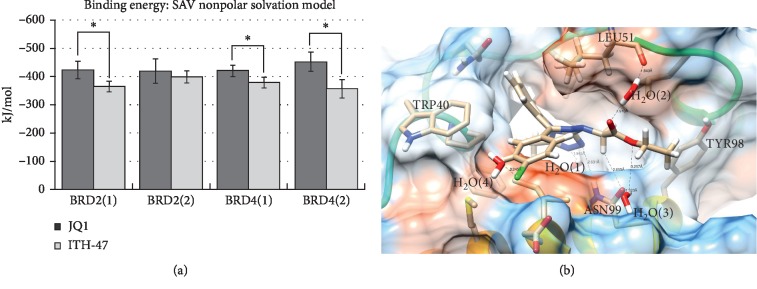
Binding energy of ITH-47 and JQ1 against BRD2(1 and 2) and BRD4(1 and 2) (a) and the binding pose of the lowest energy pose for ITH-47 against BRD4(1) (b). The binding energies of ITH-47 and JQ1 were significant for BRD2(1) and BRD4(1 and 2) (a). A low-energy conformation of the molecular dynamics simulation of ITH-47 bound to BRD4(1) revealed a ligand, protein, and water network between ASN52, LEU51, and the ligand.

Deepak et al. [28] preserved the bond angles of the X-ray and docked structures of the benzotriazepine compounds due to the fact that the bond angles from AMBER resulted in incorrect conformations of the ligands. In this study, the bond angles of the X-ray structure of JQ1 and the docked pose of ITH-47 were preserved by manually measuring the angles and consequently changing it in the ligand topology file. The molecular dynamics MMPBSA binding energy of ITH-47 was significantly less than JQ1 for BRD2(1), BRD4(1), and BRD4(2) (Figure 2(a)). The simulations show hydrogen bonds between the nitrogens of the pyrazole, ASN99, and water (H_2_O(1)) (Figure 2(b)) and the amino acid and the nitrogen pyrazoles. These interactions are conserved among benzodiazepine and benzotriazepine compounds bound to bromodomain proteins. Furthermore, we noted in a low-energy snapshot of the simulation of ITH-47 bound to BRD4(1) that the LEU51 amino acid forms a hydrogen bond with a nearby water residue which, in turn, bonds with the ester ketone of the ligand (Figure 2(b)). This was not observed in the JQ1 simulation, which may account for the selectivity of ITH-47.

### 3.2. Cell Growth

**Figure 3:**
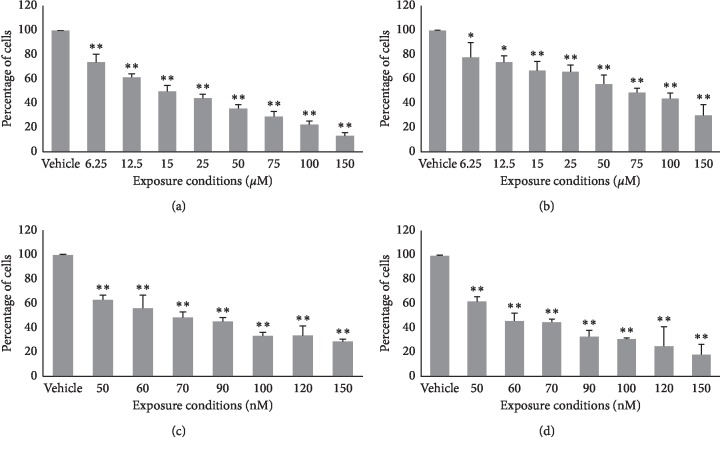
MDA-MB-231 (a and c) and MCF-7 (b and d) cell numbers expressed as a % of cells relative to the negative control (vehicle: DMSO) after being exposed to ITH-47 (6.25–150 *μ*M) (a and b) and ESE-15-ol (c and d) for 48 hours. ITH-47 inhibited the growth of MDA-MB-231 (a) and MCF-7 (b) cells by 50% (48 hours) at concentrations of 15 *μ*M (a) and 75 *μ*M (b). ESE-15-ol inhibited the growth of MDA-MB-231 (c) and MCF-7 (d) by 50% (48 hours) at concentrations of 70 nM (c) and 60 nM (d). ^*∗*^ indicates *P* value <0.05, while ^*∗∗*^ indicates *P* value <0.01 versus control.

ITH-47 concentrations ranged from 6.25 to 150 *μ*M (Figure 3(a) and 3(b)). ITH-47 suppressed cell growth in a time- and concentration-dependent manner. The GI_50_ of ITH-47 in the MDA-MB-231 cell line at 48 hours was 15 *μ*M, while, in the MCF-7 cell line, the GI_50_ of ITH-47 was 75 *μ*M. The metastatic MDA-MB-231 cell line was more sensitive to ITH-47 than the MCF-7 cell line.

ESE-15-ol concentrations ranged from 50 to 150 nM (Figures 3(c) and 3(d)). The GI_50_ of ESE-15-ol for MCF-7 and MDA-MB-231 cells at 48 hours was 60 nM and 70 nM, respectively. ESE-15-ol significantly inhibited cell growth of both MDA-MB-231 and MCF-7 cells following 48 hours of exposure.

The effect of combinations of ITH-47 and ESE-15-ol on the growth of breast cancer cells was investigated after 48 hours. Using the GI_50_ concentrations determined in the single-agent experiments, we combined ITH-47 and ESE-15-ol at 1/5x GI_50_, 2/5x GI_50_, 1/2x GI_50_, 3/5x GI_50_, 4/5x GI_50_, and GI_50_ concentrations. After exposure to a combination of 7.5 *μ*M of ITH-47 and 14 nM of ESE-15-ol for 48 hours, data revealed that the proliferation of MDA-MB-231 cells was significantly decreased when compared to single agent-exposed MDA-MB-231 cells. The CI method showed that ITH-47 and ESE-15-ol synergistically inhibited the growth of MDA-MB-231 ((CI = 0.7) Table 1).

Considering that 7.5 *μ*M of ITH-47 combined with 14 nM of ESE-15 significantly inhibited the growth of MDA-MB-231 cells, these concentrations were used to compare the effects of ITH-47 and ESE-15-ol across different cell lines.

### 3.3. Flow Cytometry: Cell Cycle Progression

**Figure 4:**
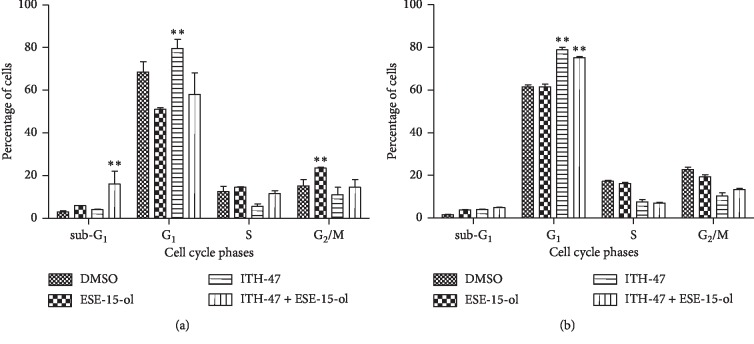
Distribution of DNA content relative to phases of the cell cycle of MDA-MB-231 cells (a) and MCF-7 cells (b). Significantly more MDA-MB-231 cells were observed in the G_1_ phase when treated with ITH-47 compared to the DMSO control and significantly more cells in the sub-G_1_ phase when treated with the ITH-47 + ESE-15-ol compared to the DMSO control following 48 hours of exposure (a). Significantly more MCF-7 cells in the G_1_ phase were observed when treated with ITH-47-treated and ITH-47 + ESE-15-ol compared to the DMSO control (b). ^*∗∗*^ indicates *P* value <0.01.

Cell cycle analysis demonstrated that MDA-MB-231 cells treated with ITH-47 alone resulted in a significant G_1_ cell cycle arrest (80%) when compared to the other treatment groups (Figure 4(a)). There were slightly more cells in the sub-G_1_ phase (3.57%) after treatment with ITH-47; however, this was not statistically significant (Figure 4(a)). MDA-MB-231 cells that were exposed to 14 nM of ESE-15-ol presented with a statistically insignificant increase in the number of cells in the sub-G_1_ phase (5.99%). Exposure of MDA-MB-231 cells to ESE-15-ol resulted in an increase in the number of cells in the G_2_/M (24%) phase in comparison with vehicle control-treated cells. Treatment with a combination of ITH-47 and ESE-15-ol resulted in significantly more MDA-MB-231 cells in the sub-G_1_ phase (18.8%) (*P* < 0.05) suggesting the induction of apoptosis.

Cell cycle analysis demonstrated that, following treatment with the combination of ITH-47 and ESE-15-ol, more MCF-7 cells were in the sub-G_1_ phase (5%) and G_1_ phase (75%) compared to vehicle-treated cells (Figure 4(b)). After treatment with only ITH-47, significantly more MCF-7 cells were in G_1_ cell cycle arrest (78%) compared to vehicle-treated cells (Figure 4(b)).

### 3.4. Flow Cytometry: Apoptosis

**Figure 5:**
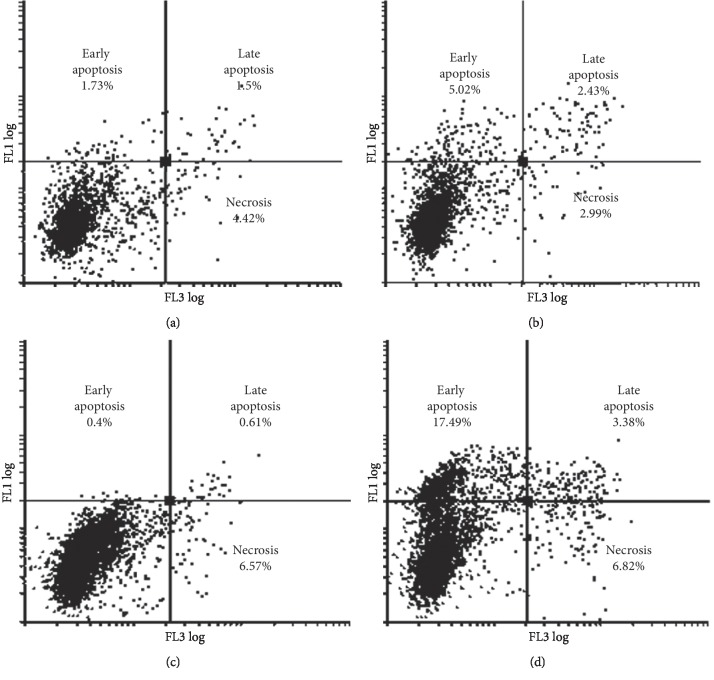
PI (FL3 log) versus annexin V (FL1 log) histogram of vehicle-treated (a), ESE-15-ol-treated (b), ITH-47-treated (c), and combination-treated (d) MDA-MB-231 cells after 48 hours of exposure. An increase in the number of cells in apoptosis (early and late stages) was observed in combination-treated cells.

**Figure 6:**
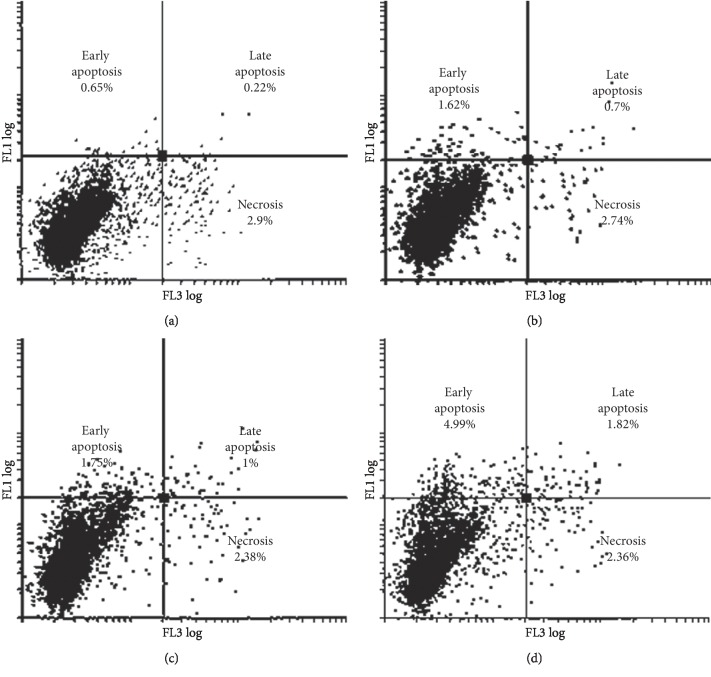
PI (FL3 log) against annexin V (FL1 log) histogram of vehicle-treated (a), ESE15-ol-treated (b), ITH-47-treated (c), and combination-treated (d) MCF-7 cells subsequent to 48 hours of treatment. Results revealed a significant rise in the number of cells present in early phases of apoptosis in combination-treated cells in comparison with the vehicle control-treated cells.

Data from both MCF-7 and MDA-MB-231 cells indicated that neither the ESE-15-ol (14 nM) nor the ITH-47 (7.5 *μ*M) alone had any significant effect in inducing apoptosis when compared to the vehicle-treated control cells (Figures 5 and 6). However, when both cell types were exposed to the combination therapy for 48 hours, there were 17.49% MDA-MB-231 and 4.99% MCF-7 cells in the early apoptotic phase. The increase was statistically significant when compared to the vehicle-treated control (*P* value <0.05) (Figures 5(d) and 6(d)). These results are in agreement with the former cell cycle analysis of sub-G_1_ results (Figure 4(a)). When compared the apoptosis induction results of the MDA-MB-231 and MCF-7 breast cancer cell lines, the differences between early apoptosis for MDA-MB-231 (17.49%) and MCF-7 (4.99%) cells were statistically significant (*P* value <0.05) (Figures 5(d) and 6(d)).

### 3.5. Spectrophotometry: Caspase 6 Colorimetric Assay

**Figure 7:**
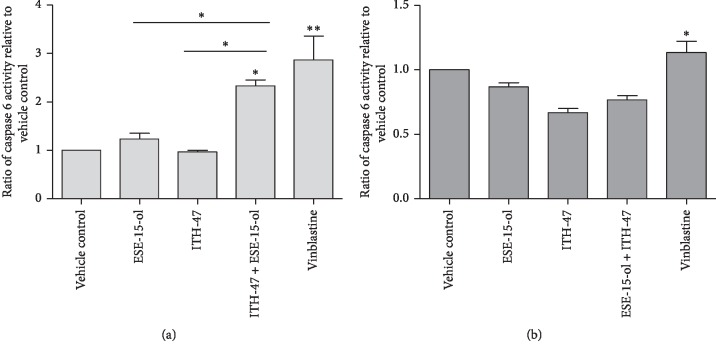
Caspase 6 activity ratios of vehicle-treated, ESE-15-ol-treated, ITH-47-treated, and combination-treated MDA-MB-231 (a) and MCF-7 (b) cells after 48 hours of exposure. Activity of caspase 6 was increased in the combination-treated MDA-MB-231 cells when compared to the vehicle-treated cells. ^*∗*^ indicates a *P* value <0.05 whereas ^*∗∗*^ indicates *P* value <0.01.

To further understand the mechanism of action by which the compounds, ITH-47 and ESE-15-ol, induce apoptosis in MDA-MB-231 and MCF-7 cells, caspase 6 colorimetric assay was conducted.  Figure 7 represents caspase 6 activity ratios of ESE-15-ol, ITH-47, and the combination-treated MDA-MB-231 and MCF-7 cells compared to vehicle-treated cells. ESE-15-ol as a single agent and in combination with ITH-47 in MDA-MB-231 resulted in a statistically significant increase in the activity of caspase 6 when compared to the vehicle-treated cells (Figure 7). Exposure of MCF-7 cells to ESE-15-ol and ITH-47 as single and in combination did not increase the activity of the executioner caspase (caspase 6) (Figure 7).

### 3.6. Protein Expression

**Figure 8:**
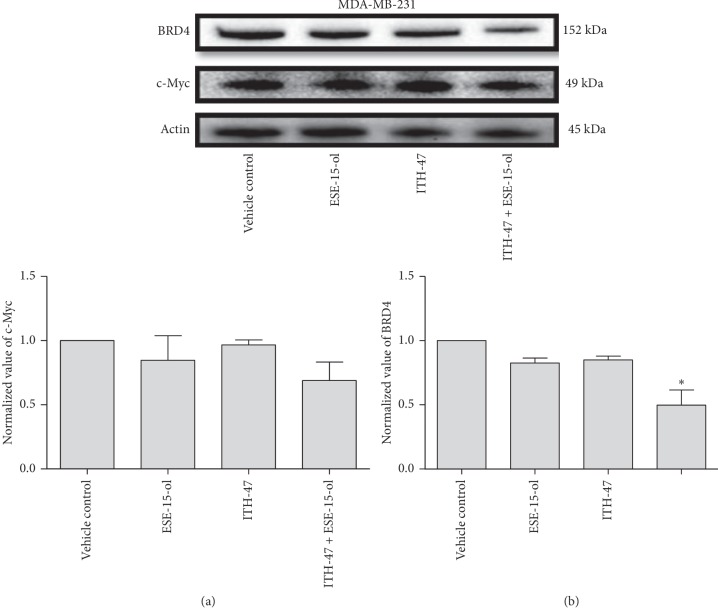
Expression of BRD-4 and c-Myc proteins. Exposure of MDA-MB-231 to ITH-47 + ESE-15-ol resulted in a decrease in both BRD4 (0.5-fold) and c-Myc (0.7-fold) protein levels when compared to all other treatment groups. ^*∗*^ indicates *P* value <0.05 while ^*∗∗*^ indicates *P* value <0.01.

In MDA-MB-231 cells, protein expression levels of BRD4 (0.83-fold; 0.85-fold) and c-Myc (0.99-fold; 0.84-fold) were not significantly different after exposure to ITH-47 and ESE-15-ol as single agents. In contrast, the combination treatment of ITH-47 and ESE-15-ol led to a decrease in BRD4 (0.5-fold) and c-Myc (0.7-fold) protein levels when compared to all other treatment groups (Figure 8).

**Figure 9:**
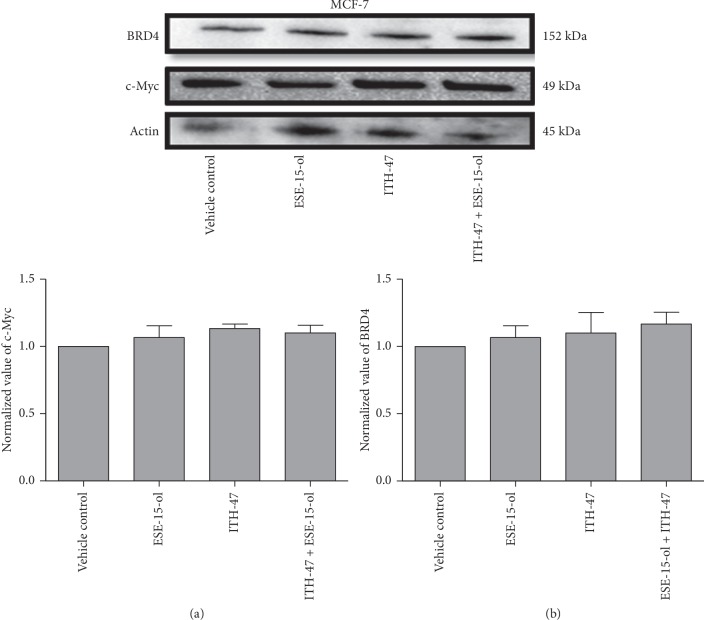
Western blot analysis of BRD-4 and c-Myc protein expression levels. Exposure of MCF-7 to ITH-47, ESE-15-ol, and combination treatment resulted in a minor increase in both BRD4 and c-Myc protein levels when compared to the vehicle-treated cells (*P* value >0.05).

Protein quantification of the MCF-7 cells revealed a statistically insignificant change in the protein expression levels of BRD4 (1.1-fold; 1.06-fold) and c-Myc (1.13-fold; 1.07-fold) when MCF-7 cells were exposed to ITH-47 and ESE-15-ol as single agents, respectively. Also, when ITH-47 was added together with ESE-15ol, the combination therapy did not significantly decrease both BRD4 (1.16-fold) and c-Myc (1.1-fold) protein levels when compared to vehicle control-treated cells (Figure 9).

## 4. Discussion

In this study, we examined if a novel drug, namely, ITH-47, which is a potential BRD4 inhibitor, combined with an antimitotic compound, namely, ESE-15-ol, would synergistically inhibit the growth of tumorigenic MCF-7 and metastatic MDA-MB-231 breast cancer cell lines.

The combination of ITH-47 and ESE-15-ol inhibited the growth of MDA-MB-231 cells, which was confirmed by the increase in the number of cells arrested in the sub-G_1_ phase of the cell cycle arrest, as well as a marked increase in apoptosis and the expression of fewer BRD4 and c-Myc proteins.

Through the use of molecular dynamics, we studied and compared the binding of ITH-47 to JQ1 (a BRD4 inhibitor). We observed a water network between the LEU51 amino acid of BRD4(1) and the ester ketone of the ITH-47 ligand. The latter was not observed in the JQ1 compound, which may be the reason why the ITH-47 has a greater affinity towards BRD4.

Although the molecular dynamics results showed that ITH-47 was less potent than JQ1, we found that 15 *μ*M of ITH-47 had a 50% growth inhibition effect on the metastatic breast cancer cell line (MDA-MB-231), whereas 75 *μ*M of ITH-47 had a 50% growth inhibition effect on the breast cancer cell line (MCF-7). In this study, the luminal MCF-7 cell line was comparably less sensitive to ITH-47 than the triple-negative MDA-MB-231 cell line. Similarly, Shu et al. reported that TNBC cancer cells were more sensitive to BET inhibition, both *in vitro* and *in vivo* [40]. In another study, Qiu et al. found that JQ1 inhibited the growth of ovarian cancer cell lines, Hey and SKOV3, despite these cell lines having functional oestrogen receptors [41]. Qiu et al. found that cells exposed to JQ1 expressed less c-Myc protein, had higher levels of apoptosis, and impaired metabolic pathways [41].

It was investigated if the potency of ITH-47 could be improved by combining it with an antimitotic drug such as ESE-15-ol. The results revealed that the two agents had a synergistic inhibitory effect on the growth of the MDA-MB-231 breast cancer cell line. By combining the compounds, the concentration of ITH-47 was reduced from 15 *μ*M to 7.5 *μ*M and that of ESE-15-ol was reduced from 70 nM to 14 nM to achieve GI_50_.

To further examine the effects of ITH-47 in combination with ESE-15-ol on MDA-MB 231 and MCF-7 cell lines, cell cycle progression studies were conducted. Results from cell cycle progression studies revealed that the treatment of both MDA-MB-231 and MCF-7 breast cancer cells to ITH-47 alone leads to a significant increase in cells present in the G_1_ phase. The results from this study were compatible with results in which it was discovered that JQ1 caused a marked G_0_/G_1_ cell cycle arrest in chondrosarcoma cells [42]. Henssen et al. showed that the exposure of medulloblastoma cell lines to JQ1 induced an increase in the quantity of cells in G_1_ phase of the cell cycle [43]. These findings suggest that BET bromodomain inhibition interrupts cells at the G_1_/S border of the cell cycle.

Furthermore, data from our cell cycle progression studies also revealed that the treatment of both MDA-MB-231 and MCF-7 breast cancer cells to ESE-15-ol alone leads to a significant increase in cells present in the G_2_/M phase. These results were consistent with previous studies from our laboratory which showed that ESE-15-ol caused a marked G_2_/M cell cycle arrest in cervical cancer (HeLa) and breast cancer (MCF-7, MDA-MB-231 and MCF-12A) cell lines [19, 20]. These results indicate that ESE-15-ol is able to induce a mitotic cell cycle arrest.

The exposure of MDA-MB-231 cells to the combination of ITH-47 and ESE-15-ol resulted in an increase in cells in the sub-G_1_ phase, which is suggestive of cells undergoing cell death via apoptosis. Contrarily, the exposure of MCF-7 cells to the combination of ITH-47 and ESE-15-ol did not significantly increase the number of cells in the sub-G_1_ phase. From these results, it was revealed that the combination resulted in a remarkable induction of apoptosis compared to exposure to each agent used individually.

Annexin V-FITC and caspase activation assays were used to further investigate the induction of apoptosis by the combination of ITH-47 and ESE15-ol in both MCF-7 and MDA-MB-231 cell lines. Annexin V-FITC results revealed that there was a statistically significant increase in the quantity of cells in early apoptosis (17.4%) and late apoptosis (7.59%) in combination-treated MDA-MB-231 cells, when compared to the vehicle control-treated cells. In MCF-7 cells, the combination of ITH-47 and ESE-15-ol did not significantly increase the number of cells in apoptosis when compared to the controls (*P* > 0.05). The inability of the combination to induce apoptosis in MCF-7 cells may be due to the lower sensitivity of these cells to ITH-47, which is an oestrogen-sensitive cell line. This finding supports our observation that significantly more ITH-47 was needed to limit cell growth in the MCF-7 cells compared with the MDA-MB-231. Our data showed that the combination of ITH-47 and ESE-15-ol induces apoptosis in MDA-MB-231 cells. These findings were compatible with a previous report which showed that 0.2 *μ*mol/l of JQ1 in combination with 0.5 *μ*mol/l of docetaxel increased the induction of apoptosis in triple-negative breast cancer cell lines (MDA-MB-231 and HS578T) within 72 hours of exposure [44].

Caspases are proteases which play a significant role in the apoptotic pathway [38]. Caspase 6 belongs to the group of executioner caspases [39]. Data from our caspase 6 studies showed that caspase 6 activity was significantly increased in combination-treated MDA-MB231 cells. However, in MCF-7 cells, the caspase 6 activity was not increased. These results suggest that caspases may be involved in the mechanism of action by which ITH-47 in combination with ESE-15-ol induces apoptosis in MDA-MB-231 cells.

Finally, we investigated the ability of ITH-47 to limit the expression of BRD4 and c-Myc protein levels in MDA-MB-231 and MCF-7 cells. As a BET inhibitor, we expected ITH-47 to decrease the production of BRD4 and c-Myc proteins, but exposure to 7.5 *μ*M ITH-47 for 48 hours had no effect on the production of these proteins. Similarly, in a study conducted by Shu et al., JQ1 did not repress c-Myc levels in TNBC cells [40, 45]. These authors proposed an epigenetic explanation for BED inhibitor resistance in TNBC cells, whereby the PP2A tumour suppressor gene is inactivated, leading to hyperphosphorylated BRD4 which binds more strongly to MED1 and allows for bromodomain-independent chromatin recruitment [40].

Shu et al. suggested that BED inhibitor resistance might be overcome by combining the inhibitor with a BCL-xL inhibitor or a CK2 inhibitor [40]. We found that combining 7.5 *μ*M of ITH-47 with 14 nM of ESE-15-ol significantly decreased both BRD4 and c-Myc protein levels, and thus observed apoptosis.

In conclusion, we have demonstrated that ITH-47 and ESE-15-ol worked synergistically to bring about antiproliferative effects in MDA-MB-231 cells by inducing apoptosis. This study is the first to investigate the *in vitro* mechanistic effect of the combination of two *in silico*-designed agents, namely, ITH-47 and ESE-15-ol in breast cancer cells. This research contributes to unravel the *in vitro* molecular mechanisms associated with a novel combination of bromodomain inhibitors and antimitotic agents, thus providing a basis for further research on these combinations.

## Figures and Tables

**Table 1:** Combination index (CI) and GI_50_ values at 48 hours for ITH-47 and ESE-15-ol combinations.

Cell line	ITH-47 (GI_50_)	ESE-15-ol(GI_50_)	Combination index	Combination GI_50_	Interpretation
MDA-MB-231	15 *μ*M	70 nM	0.7	ITH-47: 7.5 *μ*M ESE-15-ol: 14 nM	Synergism

CI < 1 indicates synergistic effect, CI = 1 indicates additive effects, and CI > 1 indicates antagonistic effect.

## Data Availability

The excel data used to support the findings of this study are available from the corresponding author upon request.

## References

[1] Brinton L. A., Figueroa J. D., Awuah B. (2014). Breast cancer in Sub-Saharan Africa: opportunities for prevention. *Breast Cancer Research and Treatment*.

[2] Demchig D., Mello-Thoms C., Brennan P. C. (2017). Breast cancer in Mongolia: an increasingly important health policy issue. *Breast Cancer: Targets and Therapy*.

[3] Bray F., Jemal A., Grey N., Ferlay J., Forman D. (2012). Global cancer transitions according to the human development index (2008–2030): a population-based study. *The Lancet Oncology*.

[4] Gerl R., Vaux D. L. (2004). Apoptosis in the development and treatment of cancer. *Carcinogenesis*.

[5] Tansey W. P. (2014). Mammalian MYC proteins and cancer. *New Journal of Science*.

[6] Mitrugno A., Sylman J. L., Ngo A. T. (2017). Aspirin therapy reduces the ability of platelets to promote colon and pancreatic cancer cell proliferation: implications for the oncoprotein c-MYC. *American Journal of Physiology-Cell Physiology*.

[7] Ngo C. V., Gee M., Akhtar N. (2000). An in vivo function for the transforming Myc protein: elicitation of the angiogenic phenotype. *Cell Growth & Differentiation: The Molecular Biology Journal of the American Association for Cancer Research*.

[8] Filippakopoulos P., Qi J., Picaud S. (2010). Selective inhibition of BET bromodomains. *Nature Reviews*.

[9] Dang C. V., Le A., Gao P. (2009). MYC-induced cancer cell energy metabolism and therapeutic opportunities. *Clinical Cancer Research*.

[10] Zuber J., Shi J., Wang E. (2011). RNAi screen identifies Brd4 as a therapeutic target in acute myeloid leukaemia. *Nature*.

[11] Kapp N., Stander X. X., Stander B. A. (2018). Synergistic in-vitro effects of combining an antiglycolytic, 3-bromopyruvate, and a bromodomain-4 inhibitor on U937 myeloid leukemia cells. *Anti-cancer Drugs*.

[12] Mukhtar E., Adhami V. M., Mukhtar H. (2014). Targeting microtubules by natural agents for cancer therapy. *Molecular Cancer Therapeutics*.

[13] Zhou J., Giannakakou P. (2005). Targeting microtubules for cancer chemotherapy. *Current Medicinal Chemistry-Anti-Cancer Agents*.

[14] Mooberry S. L., Weiderhold K. N., Dakshanamurthy S. (2007). Identification and characterization of a new tubulin-binding tetrasubstituted brominated pyrrole. *Molecular Pharmacology*.

[15] McGrogan B. T., Gilmartin B., Carney D. N., McCann A. (2008). Taxanes, microtubules and chemoresistant breast cancer. *Biochimica et Biophysica Acta (BBA)—Reviews on Cancer*.

[16] Risinger A. L., Giles F. J., Mooberry S. L. (2009). Microtubule dynamics as a target in oncology. *Cancer Treatment Reviews*.

[17] Tevaarwerk A. J., Holen K. D., Alberti D. B. (2009). Phase I trial of 2-methoxyestradiol nanocrystal dispersion in advanced solid malignancies. *Clinical Cancer Research*.

[18] Fukui M., Zhu B. T. (2009). Mechanism of 2-methoxyestradiol-induced apoptosis and growth arrest in human breast cancer cells. *Molecular Carcinogenesis*.

[19] Stander B. A., Joubert F., Tu C., Sippel K. H., McKenna R., Joubert A. M. (2012). In vitro evaluation of ESE-15-ol, an estradiol analogue with nanomolar antimitotic and carbonic anhydrase inhibitory activity. *PLoS One*.

[20] Visagie M. H., Jaiswal S. R., Joubert A. M. (2016). In vitro assessment of a computer-designed potential anticancer agent in cervical cancer cells. *Biological Research*.

[21] Blagosklonny M. V. (2004). Analysis of FDA approved anticancer drugs reveals the future of cancer therapy. *Cell Cycle*.

[22] Mokhtari R. B., Homayouni T. S., Baluch N. (2017). Combination therapy in combating cancer. *Oncotarget*.

[23] Yap T. A., Omlin A., de Bono J. S. (2013). Development of therapeutic combinations targeting major cancer signaling pathways. *Journal of Clinical Oncology*.

[24] Hass R., Bertram C. (2009). Characterization of human breast cancer epithelial cells (HBCEC) derived from long term cultured biopsies. *Journal of Experimental & Clinical Cancer Research*.

[25] Chavez K. J., Garimella S. V., Lipkowitz S. (2010). Triple negative breast cancer cell lines: one tool in the search for better treatment of triple negative breast cancer. *Breast Disease*.

[26] Pronk S., Páll S., Schulz R. (2013). Gromacs 4.5: a high-throughput and highly parallel open source molecular simulation toolkit. *Bioinformatics*.

[27] Lindorff‐Larsen K., Piana S., Palmo K. (2010). Improved side‐chain torsion potentials for the Amber ff99SB protein force field. *Proteins: Structure, Function, and Bioinformatics*.

[28] Deepak V., Wang B., Koot D. (2017). In silico design and bioevaluation of selective benzotriazepine BRD4 inhibitors with potent antiosteoclastogenic activity. *Chemical Biology & Drug Design*.

[29] Kumari R., Kumar R., Lynn A. (2014). g_mmpbsa-A GROMACS tool for high-throughput MM-PBSA calculations. *Journal of Chemical Information and Modeling*.

[30] Baker N. A., Sept D., Joseph S., Holst M. J., McCammon J. A. (2001). Electrostatics of nanosystems: application to microtubules and the ribosome. *Proceedings of the National Academy of Sciences*.

[31] Kueng W., Silber E., Eppenberger U. (1989). Quantification of cells cultured on 96-well plates. *Analytical Biochemistry*.

[32] Antoinette M. (2016). *Developing a Crystal Violet Assay to Quantify Biofilm Production Capabilities of Sstaphylococcus Aureus*.

[33] Śliwka L., Wiktorska K., Suchocki P. (2016). The comparison of MTT and CVS assays for the assessment of anticancer agent interactions. *PLoS One*.

[34] Feoktistova M., Geserick P., Leverkus M. (2016). Crystal violet assay for determining viability of cultured cells. *Cold Spring Harbor Protocols*.

[35] Chou T.-C., Talalay P. (1984). Quantitative analysis of dose-effect relationships: the combined effects of multiple drugs or enzyme inhibitors. *Advances in Enzyme Regulation*.

[36] Darzynkiewicz Z., Halicka H. D., Zhao H. (2010). Analysis of cellular DNA content by flow and laser scanning cytometry. *Advances in Experimental Medicine and Biology*.

[37] Vermes I., Haanen C., Steffens-Nakken H., Reutellingsperger C. (1995). A novel assay for apoptosis flow cytometric detection of phosphatidylserine expression on early apoptotic cells using fluorescein labelled Annexin V. *Journal of Immunological Methods*.

[38] Fan T.-J., Han L.-H., Cong R.-S., Liang J. (2005). Caspase family proteases and apoptosis. *Acta Biochimica et Biophysica Sinica*.

[39] Li J., Yuan J. (2008). Caspases in apoptosis and beyond. *Oncogene*.

[40] Shu S., Lin C. Y., He H. H. (2016). Response and resistance to BET bromodomain inhibitors in triple-negative breast cancer. *Nature*.

[41] Qiu H., Jackson A. L., Kilgore J. E. (2015). JQ1 suppresses tumor growth through downregulating LDHA in ovarian cancer. *Oncotarget*.

[42] Zhang H.-T., Gui T., Sang Y. (2017). The BET bromodomain inhibitor JQ1 suppresses chondrosarcoma cell growth via regulation of YAP/p21/c-Myc signaling. *Journal of Cellular Biochemistry*.

[43] Henssen A., Thor T., Odersky A. (2013). BET bromodomain protein inhibition is a therapeutic option for medulloblastoma. *Oncotarget*.

[44] Perez-Pena J., Serrano-Heras G., Montero J. C., Corrales-Sanchez V., Pandiella A., Ocana A. (2016). In silico analysis guides selection of BET inhibitors for triple-negative breast cancer treatment. *Molecular Cancer Therapeutics*.

[45] Sahai V., Redig A. J., Collier K. A., Eckerdt F. D., Munshi H. G. (2016). Targeting BET bromodomain proteins in solid tumors. *Oncotarget*.

